# The pyrochlore-type molybdate Pr_2_Mo_1.73_Sc_0.27_O_7_

**DOI:** 10.1107/S1600536812045138

**Published:** 2012-11-10

**Authors:** Philippe Gall, Patrick Gougeon

**Affiliations:** a Unité Sciences Chimiques de Rennes, UMR CNRS No. 6226, Université de Rennes I – INSA Rennes, Campus de Beaulieu, 35042 Rennes CEDEX, France

## Abstract

Dipraseodymium molybdenum scandium hepta­oxide, Pr_2_Mo_1.73_Sc_0.27_O_7_, crystallizes in the cubic pyrochlore-type structure. In the crystal, (Mo,Sc)O_6_ octa­hedra are linked together by common corners, forming a three-dimensional [(Mo,Sc)_2_O_6_] framework. The Pr atom and another O atom atom are located in the voids of this framework. The Mo and the Sc atom are distributed statistically over the same 16*d* crystallographic position, with site-occupancy factors of 0.867 (3) and 0.133 (3), respectively. The Pr^3+^ ions are surrounded by six O atoms from the MoO_6_ octa­hedra and by two other O atoms, forming a ditrigonal scalenohedron. All atoms lie on special positions. The Pr and the statistically distributed (Mo,Sc) sites are in the 16*c* and 16*d* positions with .-3*m* symmetry, and two O atoms are in 48*f* and 8*a* positions with 2.*mm* and -43*m* site symmetry, respectively.

## Related literature

For pyrochlore-type molybdates, see, for example: Hubert (1974 ▶); Subramanian *et al.* (1983 ▶); Gall & Gougeon (2008 ▶). For the physical properties of some rare-earth molybdate pyrochlores, see: Hill *et al.* (1989 ▶); Ali *et al.* (1989 ▶); Miyoshi *et al.* (2001 ▶, 2003 ▶). An attempt to synthesize ScPr_9_Mo_16_O_35_, a compound with the LiNd_9_Mo_16_O_35_ type structure (Gougeon *et al.*, 2011 ▶), was unsuccessful, resulting in a multiphase product with Pr_2_Mo_1.73_Sc_0.27_O_7_ and Pr_16_Mo_21_O_56_ (Gougeon & Gall, 2011 ▶) as predominant phases.

## Experimental

### 

#### Crystal data

Pr_2_Mo_1.73_Sc_0.27_O_7_
*M* *_r_* = 571.69
Cubic, 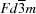

*a* = 10.5271 (3) Å
*V* = 1166.61 (6) Å^3^
*Z* = 8
Mo *K*α radiation
μ = 20.33 mm^−1^
*T* = 293 K
0.10 × 0.06 × 0.05 mm

#### Data collection

Nonius KappaCCD diffractometer
Absorption correction: analytical (de Meulenaar & Tompa, 1965 ▶) *T*_min_ = 0.302, *T*_max_ = 0.461
9208 measured reflections
326 independent reflections
269 reflections with *I* > 2σ(*I*)
*R*_int_ = 0.044

#### Refinement

*R*[*F* ^2^ > 2σ(*F* ^2^)] = 0.018
*wR*(*F* ^2^) = 0.047
*S* = 1.33
326 reflections
13 parameters
Δρ_max_ = 0.79 e Å^−3^
Δρ_min_ = −0.81 e Å^−3^

### 

Data collection: *COLLECT* (Nonius, 1998 ▶); cell refinement: *COLLECT*; data reduction: *EVALCCD* (Duisenberg *et al.*, 2003 ▶); program(s) used to solve structure: *SIR97* (Altomare *et al.*, 1999 ▶); program(s) used to refine structure: *SHELXL97* (Sheldrick, 2008 ▶); molecular graphics: *DIAMOND* (Brandenburg, 2001 ▶); software used to prepare material for publication: *SHELXL97*.

## Supplementary Material

Crystal structure: contains datablock(s) global, I. DOI: 10.1107/S1600536812045138/ru2045sup1.cif

Structure factors: contains datablock(s) I. DOI: 10.1107/S1600536812045138/ru2045Isup2.hkl

Additional supplementary materials:  crystallographic information; 3D view; checkCIF report

## Figures and Tables

**Table 1:** Selected bond lengths (Å)

Pr1—O2	2.2792
Pr1—O1^i^	2.5795 (13)
Mo1—O1^ii^	2.0440 (8)

Symmetry codes: (i) 
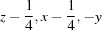
; (ii) 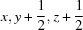.

**Fig. 1.**
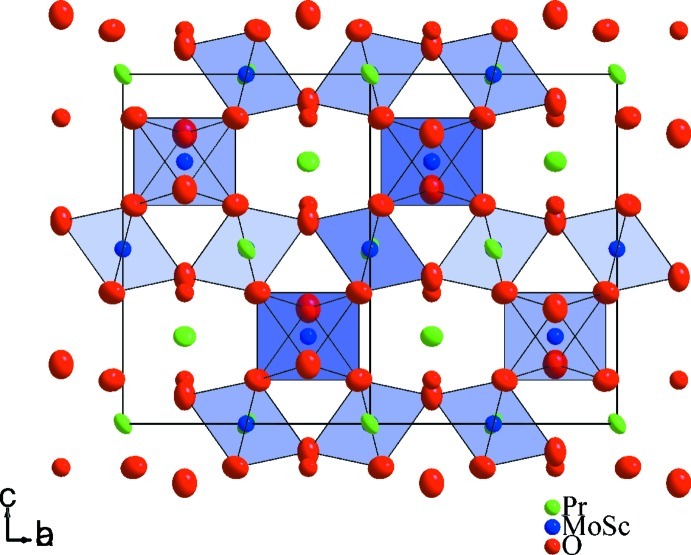
: View of Pr2Mo1.73Sc0.27O7 along the [110] direction. Displacement ellipsoids are drawn at the 97% probability level.

## supplementary crystallographic information

### Comment

An attempt to synthesize ScPr_9_Mo_16_O_35_, a compound with the LiNd_9_Mo_16_O_35_ type structure (Gougeon *et al.*, 2011), was unsuccessful, resulting in a multiphase product. However, the formation of the new compound, Pr_2_Mo_1.73_Sc_0.27_O_7_ was achieved. Recently, we presented the crystal structure of the pseudo-quaternary pyrochlore Pr_1.37_Ca_0.63_Mo_2_O_7_ (Gall & Gougeon, 2008) in which the Pr and Ca toms occupy statistically the A site. The existence of the latter two phases clearly suggests the unknown Pr_2_Mo_2_O_7_ could be synthesized. However, our attempts to obtain a single- phase powder sample as well as single-crystals of Pr_2_Mo_2_O_7_ were unsuccessful up to now.

### Experimental

Single crystals of Pr_2_Mo_1.73_Sc_0.27_O_7_ were prepared from a mixture of Pr_6_O_11_ (Rhone Poulenc, 99.99%), Sc_2_O_3_ (Strem Chemicals, 99.99%), MoO_3_ (Cerac, 99.95%) and Mo (Plansee, 99.9999%) with the nominal composition ScPr_9_Mo_16_O_35_. Before use, Mo powder was reduced under H_2_ flowing gas at 1273 K during ten hours in order to eliminate any trace of oxygen. The initial mixture (*ca* 5 g) was cold pressed and loaded into a molybdenum crucible, which was sealed under a low argon pressure using an arc welding system. The charge was heated at the rate of 300 K/h up to 2223 K, temperature which was held for 5 min., then cooled at 100 K/h down to 1373 K and finally furnace cooled. The final product was multiphasic with Pr_2_Mo_1.73_Sc_0.27_O_7_ and Pr_16_Mo_21_O_56_ (Gougeon & Gall, 2011), as predominant phases. The crystals thus obtained were of irregular shape.

### Refinement

The structure was solved by direct method using *SIR97*. The second setting, with the origin at 3 m of the Fd3m space group, was chosen. The first refinements taking into account a full occupancy of the Pr1 and Mo1 sites resulted in a *R* factor of about 0.033. Refinements of the site-occupancy factors of the Pr1 and Mo1 atoms show that the first site was fully occupied while the second one was slightly deficiency. As qualitative microanalyses using a Jeol JSM-35 CF scanning electron microscope equipped with a Tracor energy- dispersive-type X-ray spectrometer indicated the presence of scandium in the crystals, we could expect that the deficiency observed on the Mo1 site results from the presence of scandium. Refinements taking into account an occupation of the deficient Mo1 site simultaneously by Mo and Sc atoms with no constraint on the site-occupancy factors of the Mo1 and Sc1 atoms led to an occupation of 1.03 (9) of the 16 d position. Consequently, the sum of the site occupancy factors was constrained to the unity, and the ADPs of the Mo1 and Sc1 atoms were constrained to be equal. Refinement of the occupancy factor of the O2 atom in 8a position which frequently exhibits partial or total deficiency, indicates a quasi-full occupation of this position (0.97 (2) %).

### Crystal data

**Table:**

Pr_2_Mo_1.73_Sc_0.27_O_7_	Mo *K*α radiation, λ = 0.71070 Å
*M**_r_* = 571.69	Cell parameters from 5283 reflections
Cubic, *F**d*3*m*	θ = 5.5–50°
*a* = 10.5271 (3) Å	µ = 20.33 mm^−^^1^
*V* = 1166.61 (6) Å^3^	*T* = 293 K
*Z* = 8	Irregular block, black
*F*(000) = 2018	0.1 × 0.06 × 0.05 mm
*D*_x_ = 6.510 Mg m^−^^3^	

### Data collection

**Table:**

Nonius KappaCCD diffractometer	326 independent reflections
Radiation source: fine-focus sealed tube	269 reflections with *I* > 2σ(*I*)
Horizontally mounted graphite crystal monochromator	*R*_int_ = 0.044
Detector resolution: 9 pixels mm^-1^	θ_max_ = 50.0°, θ_min_ = 5.5°
φ scans (κ = 0) + additional ω scans	*h* = −19→22
Absorption correction: analytical (de Meulenaar & Tompa, 1965)	*k* = −12→22
*T*_min_ = 0.302, *T*_max_ = 0.461	*l* = −22→22
9208 measured reflections	

### Refinement

**Table:**

Refinement on *F*^2^	Primary atom site location: structure-invariant direct methods
Least-squares matrix: full	Secondary atom site location: difference Fourier map
*R*[*F*^2^ > 2σ(*F*^2^)] = 0.018	*w* = 1/[σ^2^(*F*_o_^2^) + (0.0144*P*)^2^ + 3.2607*P*] where *P* = (*F*_o_^2^ + 2*F*_c_^2^)/3
*wR*(*F*^2^) = 0.047	(Δ/σ)_max_ < 0.001
*S* = 1.33	Δρ_max_ = 0.79 e Å^−^^3^
326 reflections	Δρ_min_ = −0.81 e Å^−^^3^
13 parameters	Extinction correction: *SHELXL97* (Sheldrick, 2008), Fc^*^=kFc[1+0.001xFc^2^λ^3^/sin(2θ)]^-1/4^
0 restraints	Extinction coefficient: 0.00067 (7)

### Special details

**Table:**

Geometry. All e.s.d.'s (except the e.s.d. in the dihedral angle between two l.s. planes) are estimated using the full covariance matrix. The cell e.s.d.'s are taken into account individually in the estimation of e.s.d.'s in distances, angles and torsion angles; correlations between e.s.d.'s in cell parameters are only used when they are defined by crystal symmetry. An approximate (isotropic) treatment of cell e.s.d.'s is used for estimating e.s.d.'s involving l.s. planes.
Refinement. Refinement of *F*^2^ against ALL reflections. The weighted *R*-factor *wR* and goodness of fit *S* are based on *F*^2^, conventional *R*-factors *R* are based on *F*, with *F* set to zero for negative *F*^2^. The threshold expression of *F*^2^ > σ(*F*^2^) is used only for calculating *R*-factors(gt) *etc*. and is not relevant to the choice of reflections for refinement. *R*-factors based on *F*^2^ are statistically about twice as large as those based on *F*, and *R*- factors based on ALL data will be even larger.

### Fractional atomic coordinates and isotropic or equivalent isotropic displacement parameters (Å^2^)

**Table:**

	*x*	*y*	*z*	*U*_iso_*/*U*_eq_	Occ. (<1)
Pr1	0.0000	0.0000	0.0000	0.00874 (8)	
Mo1	0.5000	0.5000	0.5000	0.00591 (13)	0.867 (6)
Sc1	0.5000	0.5000	0.5000	0.00591 (13)	0.133 (6)
O1	0.41969 (18)	0.1250	0.1250	0.0132 (3)	
O2	0.1250	0.1250	0.1250	0.0072 (6)	0.972 (16)

### Atomic displacement parameters (Å^2^)

**Table:**

	*U*^11^	*U*^22^	*U*^33^	*U*^12^	*U*^13^	*U*^23^
Pr1	0.00874 (8)	0.00874 (8)	0.00874 (8)	−0.00193 (2)	−0.00193 (2)	−0.00193 (2)
Mo1	0.00591 (13)	0.00591 (13)	0.00591 (13)	−0.00007 (4)	−0.00007 (4)	−0.00007 (4)
Sc1	0.00591 (13)	0.00591 (13)	0.00591 (13)	−0.00007 (4)	−0.00007 (4)	−0.00007 (4)
O1	0.0175 (7)	0.0110 (4)	0.0110 (4)	0.000	0.000	−0.0014 (5)
O2	0.0072 (6)	0.0072 (6)	0.0072 (6)	0.000	0.000	0.000

### Geometric parameters (Å, º)

**Table:**

Pr1—O2^i^	2.2792	Mo1—O1^xiii^	2.0440 (8)
Pr1—O2	2.2792	Mo1—O1^xiv^	2.0440 (8)
Pr1—O1^ii^	2.5795 (13)	Mo1—O1^xv^	2.0440 (8)
Pr1—O1^iii^	2.5795 (13)	Mo1—O1^xvi^	2.0440 (8)
Pr1—O1^iv^	2.5795 (13)	O1—Sc1^xvii^	2.0440 (8)
Pr1—O1^v^	2.5795 (13)	O1—Mo1^xvii^	2.0440 (8)
Pr1—O1^vi^	2.5795 (13)	O1—Sc1^xviii^	2.0440 (8)
Pr1—O1^vii^	2.5795 (13)	O1—Mo1^xviii^	2.0440 (8)
Pr1—Pr1^v^	3.7219 (1)	O1—Pr1^v^	2.5795 (13)
Pr1—Pr1^viii^	3.7219 (1)	O1—Pr1^viii^	2.5795 (13)
Pr1—Pr1^ix^	3.7219 (1)	O2—Pr1^viii^	2.2792
Pr1—Pr1^x^	3.7219 (1)	O2—Pr1^v^	2.2792
Mo1—O1^xi^	2.0440 (8)	O2—Pr1^x^	2.2792
Mo1—O1^xii^	2.0440 (8)		
			
O2^i^—Pr1—O2	180.0	O1^vi^—Pr1—Pr1^ix^	43.83 (3)
O2^i^—Pr1—O1^ii^	79.09 (3)	O1^vii^—Pr1—Pr1^ix^	136.17 (3)
O2—Pr1—O1^ii^	100.91 (3)	Pr1^v^—Pr1—Pr1^ix^	180.0
O2^i^—Pr1—O1^iii^	100.91 (3)	Pr1^viii^—Pr1—Pr1^ix^	120.0
O2—Pr1—O1^iii^	79.09 (3)	O2^i^—Pr1—Pr1^x^	144.7
O1^ii^—Pr1—O1^iii^	180.00 (6)	O2—Pr1—Pr1^x^	35.3
O2^i^—Pr1—O1^iv^	79.09 (3)	O1^ii^—Pr1—Pr1^x^	82.59 (3)
O2—Pr1—O1^iv^	100.91 (3)	O1^iii^—Pr1—Pr1^x^	97.41 (3)
O1^ii^—Pr1—O1^iv^	116.506 (19)	O1^iv^—Pr1—Pr1^x^	136.17 (3)
O1^iii^—Pr1—O1^iv^	63.494 (19)	O1^v^—Pr1—Pr1^x^	43.83 (3)
O2^i^—Pr1—O1^v^	100.91 (3)	O1^vi^—Pr1—Pr1^x^	82.59 (3)
O2—Pr1—O1^v^	79.09 (3)	O1^vii^—Pr1—Pr1^x^	97.41 (3)
O1^ii^—Pr1—O1^v^	63.494 (19)	Pr1^v^—Pr1—Pr1^x^	60.0
O1^iii^—Pr1—O1^v^	116.506 (19)	Pr1^viii^—Pr1—Pr1^x^	60.0
O1^iv^—Pr1—O1^v^	180.00 (6)	Pr1^ix^—Pr1—Pr1^x^	120.0
O2^i^—Pr1—O1^vi^	79.09 (3)	O1^xi^—Mo1—O1^xii^	83.22 (6)
O2—Pr1—O1^vi^	100.91 (3)	O1^xi^—Mo1—O1^xiii^	96.78 (6)
O1^ii^—Pr1—O1^vi^	116.506 (19)	O1^xii^—Mo1—O1^xiii^	180.0
O1^iii^—Pr1—O1^vi^	63.494 (19)	O1^xi^—Mo1—O1^xiv^	83.22 (6)
O1^iv^—Pr1—O1^vi^	116.506 (19)	O1^xii^—Mo1—O1^xiv^	96.78 (6)
O1^v^—Pr1—O1^vi^	63.494 (19)	O1^xiii^—Mo1—O1^xiv^	83.22 (6)
O2^i^—Pr1—O1^vii^	100.91 (3)	O1^xi^—Mo1—O1^xv^	96.78 (6)
O2—Pr1—O1^vii^	79.09 (3)	O1^xii^—Mo1—O1^xv^	83.22 (6)
O1^ii^—Pr1—O1^vii^	63.494 (19)	O1^xiii^—Mo1—O1^xv^	96.78 (6)
O1^iii^—Pr1—O1^vii^	116.506 (19)	O1^xiv^—Mo1—O1^xv^	180.0
O1^iv^—Pr1—O1^vii^	63.494 (19)	O1^xi^—Mo1—O1^xvi^	180.0
O1^v^—Pr1—O1^vii^	116.506 (19)	O1^xii^—Mo1—O1^xvi^	96.78 (6)
O1^vi^—Pr1—O1^vii^	180.00 (6)	O1^xiii^—Mo1—O1^xvi^	83.22 (6)
O2^i^—Pr1—Pr1^v^	144.7	O1^xiv^—Mo1—O1^xvi^	96.78 (6)
O2—Pr1—Pr1^v^	35.3	O1^xv^—Mo1—O1^xvi^	83.22 (6)
O1^ii^—Pr1—Pr1^v^	82.59 (3)	Sc1^xvii^—O1—Mo1^xvii^	0.0
O1^iii^—Pr1—Pr1^v^	97.41 (3)	Sc1^xvii^—O1—Sc1^xviii^	131.13 (9)
O1^iv^—Pr1—Pr1^v^	82.59 (3)	Mo1^xvii^—O1—Sc1^xviii^	131.13 (9)
O1^v^—Pr1—Pr1^v^	97.41 (3)	Sc1^xvii^—O1—Mo1^xviii^	131.13 (9)
O1^vi^—Pr1—Pr1^v^	136.17 (3)	Mo1^xvii^—O1—Mo1^xviii^	131.13 (9)
O1^vii^—Pr1—Pr1^v^	43.83 (3)	Sc1^xviii^—O1—Mo1^xviii^	0.0
O2^i^—Pr1—Pr1^viii^	144.7	Sc1^xvii^—O1—Pr1^v^	106.64 (2)
O2—Pr1—Pr1^viii^	35.3	Mo1^xvii^—O1—Pr1^v^	106.64 (2)
O1^ii^—Pr1—Pr1^viii^	136.17 (3)	Sc1^xviii^—O1—Pr1^v^	106.64 (2)
O1^iii^—Pr1—Pr1^viii^	43.83 (3)	Mo1^xviii^—O1—Pr1^v^	106.64 (2)
O1^iv^—Pr1—Pr1^viii^	82.59 (3)	Sc1^xvii^—O1—Pr1^viii^	106.64 (2)
O1^v^—Pr1—Pr1^viii^	97.41 (3)	Mo1^xvii^—O1—Pr1^viii^	106.64 (2)
O1^vi^—Pr1—Pr1^viii^	82.59 (3)	Sc1^xviii^—O1—Pr1^viii^	106.64 (2)
O1^vii^—Pr1—Pr1^viii^	97.41 (3)	Mo1^xviii^—O1—Pr1^viii^	106.64 (2)
Pr1^v^—Pr1—Pr1^viii^	60.0	Pr1^v^—O1—Pr1^viii^	92.34 (6)
O2^i^—Pr1—Pr1^ix^	35.3	Pr1—O2—Pr1^viii^	109.5
O2—Pr1—Pr1^ix^	144.7	Pr1—O2—Pr1^v^	109.5
O1^ii^—Pr1—Pr1^ix^	97.41 (3)	Pr1^viii^—O2—Pr1^v^	109.5
O1^iii^—Pr1—Pr1^ix^	82.59 (3)	Pr1—O2—Pr1^x^	109.5
O1^iv^—Pr1—Pr1^ix^	97.41 (3)	Pr1^viii^—O2—Pr1^x^	109.5
O1^v^—Pr1—Pr1^ix^	82.59 (3)	Pr1^v^—O2—Pr1^x^	109.5

Symmetry codes: (i) −*x*, −*y*, −*z*; (ii) *z*−1/4, *x*−1/4, −*y*; (iii) −*z*+1/4, −*x*+1/4, *y*; (iv) *x*−1/4, *y*−1/4, −*z*; (v) −*x*+1/4, −*y*+1/4, *z*; (vi) −*y*, *z*−1/4, *x*−1/4; (vii) *y*, −*z*+1/4, −*x*+1/4; (viii) *y*+1/4, −*x*, *z*+1/4; (ix) −*x*−1/4, −*y*−1/4, *z*; (x) *x*, −*y*+1/4, −*z*+1/4; (xi) −*y*+1/2, −*z*+1/2, −*x*+1; (xii) *x*, *y*+1/2, *z*+1/2; (xiii) −*x*+1, −*y*+1/2, −*z*+1/2; (xiv) *z*+1/2, *x*, *y*+1/2; (xv) −*z*+1/2, −*x*+1, −*y*+1/2; (xvi) *y*+1/2, *z*+1/2, *x*; (xvii) *x*, −*y*+3/4, −*z*+3/4; (xviii) *x*, *y*−1/2, *z*−1/2.
